# Inhibition of Mitochondrial Uncoupling Proteins Arrests Human Spermatozoa Motility without Compromising Viability

**DOI:** 10.3390/antiox12020409

**Published:** 2023-02-08

**Authors:** David F. Carrageta, Laís Freire-Brito, Bárbara Guerra-Carvalho, João C. Ribeiro, Bruno S. Monteiro, Alberto Barros, Pedro F. Oliveira, Mariana P. Monteiro, Marco G. Alves

**Affiliations:** 1 Endocrine and Metabolic Research, UMIB—Unit for Multidisciplinary Research in Biomedicine, ICBAS—School of Medicine and Biomedical Sciences, University of Porto, 4050-313 Porto, Portugal; 2 Laboratory of Physiology, Department of Immuno-Physiology and Pharmacology, ICBAS—School of Medicine and Biomedical Sciences, University of Porto, 4050-313 Porto, Portugal; 3 Laboratory for Integrative and Translational Research in Population Health (ITR), University of Porto, 4200-465 Porto, Portugal; 4 Associated Laboratory for Green Chemistry (LAQV) of the Network of Chemistry and Technology (REQUIMTE), Department of Chemistry, University of Aveiro, 3810-193 Aveiro, Portugal; 5 Department of Pathology, Faculty of Medicine, University of Porto, 4200-319 Porto, Portugal; 6 i3S—Instituto de Investigação e Inovação em Saúde, University of Porto, 4200-135 Porto, Portugal; 7 Centre for Reproductive Genetics Prof. Alberto Barros, 4100-012 Porto, Portugal

**Keywords:** genipin, mitochondria, mitochondrial uncoupling proteins, motility, oxidative stress, spermatozoa, UCPs

## Abstract

Mitochondrial uncoupling proteins (UCPs) are central in the regulation of mitochondrial activity and reactive oxygen species (ROS) production. High oxidative stress is a major cause of male infertility; however, UCPs expression and function in human spermatozoa are still unknown. Herein, we aimed to assess the expression and function of the different homologs (UCP1-6) in human spermatozoa. For this purpose, we screened for the mRNA expression of all UCP homologs. Protein expression and immunolocalization of UCP1, UCP2, and UCP3 were also assessed. Highly motile spermatozoa were isolated from human normozoospermic seminal samples (n = 16) and incubated with genipin, an inhibitor of UCPs (0, 0.5, 5, and 50 µM) for 3 h at 37 °C. Viability and total motility were assessed. Mitochondrial membrane potential and ROS production were evaluated. Media were collected and the metabolic profile and antioxidant potential were analyzed by ^1^H-NMR and FRAP, respectively. The expression of all UCP homologs (*UCP1-6*) mRNA by human spermatozoa is herein reported for the first time. UCP1-3 are predominant at the head equatorial segment, whereas UCP1 and UCP2 are also expressed at the spermatozoa midpiece, where mitochondria are located. The inhibition of UCPs by 50 µM genipin, resulting in the UCP3 inhibition, did not compromise sperm cell viability but resulted in irreversible total motility loss that persisted despite washing or incubation with theophylline, a cAMP activator. These effects were associated with decreased mitochondrial membrane potential and lactate production. No differences concerning UCP3 expression, however, were observed in spermatozoa from normozoospermic versus asthenozoospermic men (n = 6). The inhibition of UCPs did not increase ROS production, possibly due to the decreased mitochondrial activity and genipin antioxidant properties. In sum, UCPs are major regulators of human spermatozoa motility and metabolism. The discovery and characterization of UCPs’ role in human spermatozoa can shed new light on spermatozoa ROS-related pathways and bioenergetics physiology.

## 1. Introduction

Mitochondrial uncoupling proteins (UCPs) are transmembrane proteins of the mitochondrial anionic transporter family SLC25. Six homologs of mammalian UCPs (UCP1-6) have been so far identified [1]. UCPs are expressed in the inner mitochondrial membrane, where they act as central regulators of mitochondrial membrane potential, oxidative phosphorylation, and reactive oxygen species (ROS) production [2,3]. UCPs have been known for their uncoupling activity, although this feature is absent in some of the homologs. UCP1, also known as thermogenin, is the main uncoupling homolog responsible for dissipating the proton gradient for heat production, i.e., thermogenesis. Compared with UCP1, the remaining homologs (UCP2-6) either lack or exhibit less proton transport and uncoupling function [4]. Nevertheless, all the homologs share the common characteristic of acting as regulators of redox balance, through regulation of ROS production [1,2]. This highlights a possible role for UCPs in male fertility, as high levels of oxidative stress are recognized as a major cause of infertility. Alarmingly, a temporal trend for a decrease in sperm counts was reported, leading to poorer semen quality and decreased male fertility worldwide [5]. The decline in semen quality is positively linked to oxidative stress, as it is estimated that up to 80% of infertile men can have high ROS levels, which led to a novel terminology being proposed of Male Oxidative Stress Infertility (MOSI) [6]. The origin of increased ROS production and oxidative stress is multifactorial. For instance, metabolic diseases, inflammation, exposure to certain drugs and pollutants, and ageing are all known factors that stimuli ROS production [7,8,9,10]. Oxidative stress leads to a myriad of harmful and irreversible consequences for male fertility, including impaired spermatogenesis, decreased spermatozoa concentration, and decreased numbers of motile spermatozoa [11]. The most highlighted phenomena associated with ROS are lipid peroxidation and consequently, reduced membrane fluidity [12], and defective axonemal phosphorylation [13]. It should also be noticed that DNA fragmentation and genomic instability are common traits of oxidative stress-induced damage in spermatozoa [14], which is linked to impaired oocyte fertilization, abnormal embryo development, and, ultimately, increased miscarriage rates [15,16,17]. Due to their activity on redox homeostasis, we hypothesized that UCPs could be a relevant target to understand MOSI and improve male reproductive outcomes. Although UCPs are widely expressed throughout the human body, data concerning their expression and role in the male gametes and reproductive system remain elusive. In this study, we aimed to characterize the expression and function of UCPs in human spermatozoa. For this purpose, mRNA expression of UCP 1 to 6 homologs by human spermatozoa was screened. Spermatozoa expression and location of UCP1, UCP2, and UCP3 proteins, using commercially available and reliable antibodies, was also assessed. To study the function of UCPs in human spermatozoa, we used the UCPs inhibitor genipin [18]. The effect of UCPs inhibition on human spermatozoa viability and motility was evaluated. In addition, the effect of UCPs inhibition on human spermatozoa mitochondrial membrane potential, total endogenous ROS production, as well as the spermatozoa culture media total antioxidant capacity and metabolic profile, were also evaluated.

## 2. Materials and Methods

### 2.1. Reagents and Chemicals

Unless stated otherwise, all reagents and chemicals were purchased from Sigma-Aldrich (St. Louis, MO, USA).

### 2.2. Patient Recruitment and Seminal Sample Preparation

**Figure 1:**
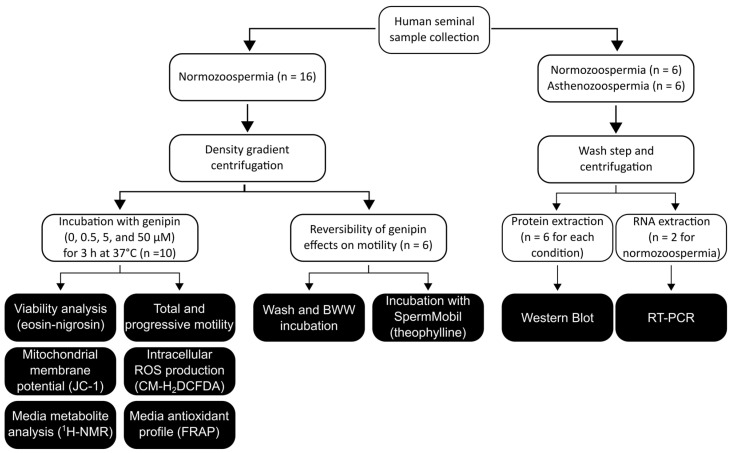
Schematic workflow of the experimental design applied in this study.

Human spermatozoa were collected from patients attending the Medical Assisted Procreation Center, after approval by the Joint Ethics Committee CHUP/ICBAS (2021/CE/P002[P342/CETI/ICBAS]). Patient recruitment, clinical evaluation, and diagnostic semen analysis were performed at the clinic in compliance with the standards of care. All procedures were conducted according to the ethical guidelines for human research, under strict individual anonymity, and informed and written consent was obtained from all participants included in the study. Men with a history of abusing drugs or alcoholism who were under treatment or exposed to drugs known to interfere with fertility, who were diagnosed with an acute genital inflammatory disease before sperm collection, who were a survivor of cancer treatment, or who had any systemic disease known to affect fertility, were excluded from the study. Smoking was not an exclusion criterion applied in this study. Sperm samples from otherwise healthy adult males were obtained by masturbation into specific sterile containers after 2 to 4 days of sexual abstinence, left at 37 °C until complete liquefaction, then analyzed according to the guidelines of the World Health Organization (WHO) [19]. Adult male subjects, normozoospermic (n = 16, mean age 38.3 ± 7.2) or asthenozoospermic (total motility < 40%, n = 6, mean age 36.6 ± 3.8), were enrolled in this study (Figure 1). A standard discontinuous density gradient centrifugation protocol was performed on 16 selected samples of normozoospermic men using the Gems*^®^* Sperm Wash Gradient Set (45% and 90%) (SWG-01, Genea, Sydney, Australia) according to the manufacturer’s instructions. The resultant pellet, containing the spermatozoa fraction with a higher percentage of motility and viability, was collected and washed two times in phosphate buffer saline (PBS) followed by centrifugation of 500× *g* for 5 min before being used for subsequent experiments. The asthenozoospermic samples and a fraction of the whole normozoospermic samples (n = 6 for each condition) were washed two times in PBS, centrifuged, and the pellet was collected for protein analysis.

### 2.3. Reverse Transcriptase-Polymerase Chain Reaction (RT-PCR)

Total RNA was extracted from pelleted spermatozoa of 2 normozoospermic men using the NZY Total RNA Isolation Kit (MB13402, NZYTech, Lisbon, Portugal) and according to the manufacturer’s instruction. Purified RNA concentration was determined using a Take3 microvolume plate on a Synergy™ H1 multi-mode microplate reader (BioTek, Winooski, VT, USA). RNA was reversely transcribed using the NZY M-MuLV Reverse Transcriptase Kit (MB083, NZYTech, Lisbon, Portugal). RT-PCR was performed to identify the expression of *UCP1*, *UCP2*, *UCP3* (*UCP3S* and *UCP3L* for short and long isoforms, respectively), *UCP4*, *UCP5*, and *UCP6* mRNAs in human spermatozoa using the resulting complementary DNA (cDNA) and specific primer sets (Table 1). Peripheral blood lymphocytes (PBL) cDNA was used as a positive control.

### 2.4. Western Blot

Proteins from pelleted human spermatozoa were extracted using radioimmunoprecipitation assay (RIPA) buffer and then quantified using Pierce™ BCA Protein Assay Kit (Thermo Fischer Scientific, Waltham, MA, USA). Western Blot protocol was performed to identify the expression of UCP1, UCP2, and UCP3 using standard methods [20]. In brief, protein samples (30 µg) were denatured at 95 °C for 5 min (UCP3) or 70 °C for 10 min (UCP1 and UCP2). Protein samples were fractionated on a 12.5% polyacrylamide gel from TGX Stain-Free™ FastCast™ Acrylamide Kit (Bio-Rad, Hemel Hempstead, UK) and transferred to a low fluorescence polyvinylidene difluoride (PVDF) membrane (Bio-Rad, Hemel Hempstead, UK) using a Trans-Blot^®^ Turbo™ System (Bio-Rad, Hemel Hempstead, UK). Membranes were blocked with a solution of 5% non-fat milk in Tris-buffered saline with 0.1% Tween-20 solution. Then, membranes were incubated overnight at 4 °C separately with primary antibodies rabbit anti-UCP1 (1:500, ab155117, Abcam, Cambridge, UK), rabbit anti-UCP2 (1:1000, PA5-77553, Invitrogen, Waltham, MA, USA), or rabbit anti-UCP3 (1:500, ab180643, Abcam, Cambridge, UK). Proteins were detected upon incubation with goat anti-rabbit IgG-AP (1:5000, A3687, Sigma-Aldrich, St. Louis, MO, USA) and reacted with ECF Substrate (RPN5785, Cytiva, Marlborough, MA, USA). Total protein content was evaluated using Ponceau S staining. Protein bands were detected using a Bio-Rad ChemiDoc XR (Bio-Rad, Hemel Hempstead, UK).

### 2.5. Immunofluorescence Staining

Immunofluorescence staining was performed to observe the cellular location of UCP1, UCP2, and UCP3 using standard methods [21]. In brief, human spermatozoa were fixed on glass coverslips with 4% paraformaldehyde in PBS for 20 min at room temperature. Spermatozoa were permeabilized with 0.1% Triton X-100 in PBS and blocked in 0.1% gelatin in PBS, both for 15 min at room temperature. Spermatozoa were then incubated with primary antibodies rabbit anti-UCP1 (1:50), rabbit anti-UCP2 (1:50), or rabbit anti-UCP3 (1:50) overnight at 4 °C. Spermatozoa incubated without primary antibodies were used as a negative control. Incubation with the secondary antibody Alexa Fluor 488 goat anti-rabbit IgG (1:1500, A-11008, Thermo Fischer Scientific, Waltham, MA, USA) occurred for 1 h at room temperature. Coverslips were mounted and nuclei were stained with VECTASHIELD^®^ Antifade Mounting Medium with DAPI (Vector Laboratories, Burlingame, CA, USA). Fluorescence was observed in a Nikon Eclipse E400 microscope equipped with a DS-Fi3 microscope camera, a Y-FL Epi-Fluorescence Attachment, and HB-10103AF Super High-Pressure Mercury Lamp Power Supply (Nikon, Shinagawa, Tokyo, Japan). The pictures obtained were processed using the NIS-Elements Br 4.60.00 software (Nikon, Shinagawa, Tokyo, Japan).

### 2.6. Experimental Groups

To study the role of UCPs in human spermatozoa physiology, we used genipin (Acros Organics™, Thermo Fisher Scientific, Waltham, MA, USA), a known UCPs inhibitor [18]. Retrieved spermatozoa from each sample (n = 10) were isolated through density gradient centrifugation and allocated into four groups (3 million spermatozoa per condition). Spermatozoa were incubated at 37 °C in 1 mL of modified Biggers, Whitter, and Whittingham (BWW) medium (in mM: 94.5 NaCl, 4.8 KCl, 1.7 CaCl_2_, 1.17 KH_2_PO_4_, 1.22 MgSO_4_, 20 HEPES, 25 NaHCO_3_, 11 Glucose, pH = 7.4) in the absence (control group) or supplemented with increasing concentrations of genipin (0.5, 5, and 50 µM). The doses of genipin were selected based on studies reported in the literature. The highest concentration used in our study (50 µM) was chosen based on the IC_50_ value reported for UCP3 inhibition (42 ± 12 µM) (13). A pilot study was performed (data not shown) using an even higher concentration (100 µM, based on the IC_50_ value reported for UCP2 inhibition—94 ± 24 µM (13)) but since total loss of motility was observed with 50 µM of genipin, we decided to exclude this concentration from further studies. The lower doses of genipin (0.5 and 5 µM) were chosen according to the mitochondrial and metabolic effects previously reported (33). To avoid the formation of genipin–albumin complexes, spermatozoa were incubated in the absence of bovine serum albumin (BSA) [22]. After 3 h incubation, samples were centrifuged and culture media were collected for Proton Nuclear Magnetic Resonance (^1^H-NMR) and Ferric Reducing Antioxidant Power Assay (FRAP) analysis. Spermatozoa were washed two times in PBS and a fraction from each condition was collected for viability, motility, mitochondrial membrane potential, or intracellular ROS content analysis.

### 2.7. Spermatozoa Viability and Motility Analysis

The effects of UCPs inhibition in human spermatozoa were assessed using eosin–nigrosin staining, according to an established protocol [23]. The percentage of viable and nonviable spermatozoa was determined by counting a total of 100 spermatozoa per slide in continuous random fields under a bright-field optical microscope (1000× magnification). White spermatozoa were considered viable whereas pink-stained spermatozoa, indicating loss of membrane integrity, were considered nonviable. Results are expressed as percentage of viable spermatozoa.

Spermatozoa total and progressive motility were assessed using a Makler counting chamber (Sefi Medical Instruments, Haifa, Israel), as previously described [23]. Spermatozoa total and progressive motility were evaluated using 10 s videos recorded in a bright-field optical microscope (200× magnification). The experiment was performed by the same user and the same criteria were applied between samples. New drops were placed at the chamber and the process was repeated until a minimum total of 100 sperm cells per sample were counted. Results are expressed as percentage of total or progressive motile spermatozoa.

### 2.8. Genipin Effects on Spermatozoa Motility

Two approaches were used to assess whether the effects of genipin-induced UCPs inhibition in spermatozoa motility were reversible (n = 6 for each procedure). In the first one, genipin-treated spermatozoa were washed twice in PBS and incubated in fresh BWW medium supplemented with BSA (0.4 mM) for 45 min at 37 °C. In the second approach, the cAMP activator theophylline, was used under the formulation of SpermMobil (GM501, Gynemed, Lensahn, Germany). SpermMobil is clinically used for the in vitro examination of immotile spermatozoa isolated from testicular tissue, as it confers motility to otherwise immotile spermatozoa through the activation of cAMP-dependent pathways. The procedure was conducted according to the manufacturer’s instructions. In brief, genipin-treated spermatozoa were washed twice in PBS and incubated in fresh BWW medium supplemented with SpermMobil medium (1:10 dilution, v:v) for 20 min at 37 °C. After each procedure, total and progressive motility were evaluated and reported as described in Section 2.7.

### 2.9. JC-1 Assay for Mitochondrial Membrane Potential Analysis

The mitochondrial membrane potential of spermatozoa was analyzed using the fluorogenic dye JC-1 (T3168, Invitrogen™, Carlsbad, CA, USA), according to a protocol previously established [24]. Briefly, 10^6^ spermatozoa from each condition were washed and collected after treatments and incubated in a JC-1 working solution (1 µg/mL in PBS) at 37 °C for 30 min. Spermatozoa incubated at 20% DMSO solution were used as an assay positive control. Spermatozoa were then transferred into a 96-well for the assessment of JC-1 monomers (485/530 nm; excitation/emission) and J-aggregates (535/590 nm; excitation/emission) fluorescence on a Synergy™ H1 multi-mode microplate reader (BioTek, Winooski, VT, USA). The ratio between JC-1 J-aggregates/monomers was calculated and used as surrogate of mitochondrial membrane polarization. Results are expressed as mean J-aggregates/monomers ratio.

### 2.10. Detection of Intracellular Reactive Oxygen Species (ROS)

The total intracellular ROS production of spermatozoa was quantified using the fluorogenic dye CM-H_2_DCFDA (C6827, Invitrogen™, Carlsbad, CA, USA). The protocol was performed as previously described [23]. Briefly, 10^5^ spermatozoa were washed and collected after treatments and incubated in a CM-H_2_DCFDA working solution (5 µM in PBS) at 37 °C for 30 min. Spermatozoa incubated in a 0.1% H_2_O_2_ solution were used as a positive control. Spermatozoa were then transferred into a 96-well black plate and fluorescence (495/529 nm; excitation/emission) was read on a Synergy™ H1 multi-mode microplate reader (BioTek, Winooski, VT, USA). Results are represented in DCF Fluorescence Units (arbitrary units).

### 2.11. Proton Nuclear Magnetic Resonance (^1^H-NMR)

The proton nuclear magnetic resonance (^1^H-NMR) spectra were acquired using a Bruker Avance III HD 500 MHz spectrometer equipped with a 5-mm TXI probe (Bruker Corporation, Billerica, MA, USA). Solvent-suppressed ^1^H-NMR spectra were acquired at 298K with a zgpr pulse with a sweep width of 7 kHz, using a relaxation delay of 7s, a pulse angle of 30°, an acquisition time of 2.3 s, and 32 scans. Before Fourier transform, each free induction decay was multiplied by a 0.2 Hz Lorentzian. A total of 180 µL of the medium of each condition was analyzed using 3-mm NMR tubes and optimizer inserts for 5-mm spinners. Samples were diluted with sodium fumarate (final concentration of 2 mM) in deuterated water solution (final volume of 225 µL), which was used as an internal reference (6.5 ppm) to quantify the following metabolites present at the medium (multiplet, ppm): lactate (doublet, 1.33) and acetate (singlet, 1.90). Spectra were manually phased and baseline corrected. Chosen metabolite peaks were integrated using the NUTS-Pro NMR software (Acorn NMR, Inc., Fremont, CA, USA). Metabolite variation (consumption/production) in each condition was determined and the results are expressed as nmol/10^6^ spermatozoa.

### 2.12. Ferric-Reducing Antioxidant Power Assay (FRAP)

The ferric-reducing antioxidant power assay (FRAP) was performed according to the method described by Benzie and Strain [25]. Culture media was added to the working FRAP solution, which reduces the Fe^3+^-TPTZ complex to the colored Fe^2+^-TPTZ complex. The experiment was performed in duplicates for each sample. After 40 min incubation, the absorbance was read at 593 nm on a Synergy™ H1 multi-mode microplate reader (BioTek, Winooski, VT, USA). Dilutions of ascorbic acid (0–2 µM) were used for the standard curve. FRAP value of the media was calculated as described by Benzie and Strain [25]. Results are expressed as µmol of antioxidant potential/L.

### 2.13. Statistical Analysis

Experimental results are represented as mean ± standard deviation (SD) or Tukey’s boxplot (median, 25th to 75th percentiles ± 1.5 IQR). Statistical analysis was performed in GraphPad Prism 9 (GraphPad Software, San Diego, CA, USA). Data were tested for normal distribution with the Kolmogorov–Smirnov test. Statistical differences were determined by a paired *t*-test or repeated measures one-way ANOVA with post hoc Dunnett’s multiple comparisons test. *p* < 0.05 was considered significantly different.

## 3. Results

### 3.1. UCP1-6 mRNAs and UCP1, UCP2, and UCP3 Proteins Are Present in Human Spermatozoa

**Figure 2:**
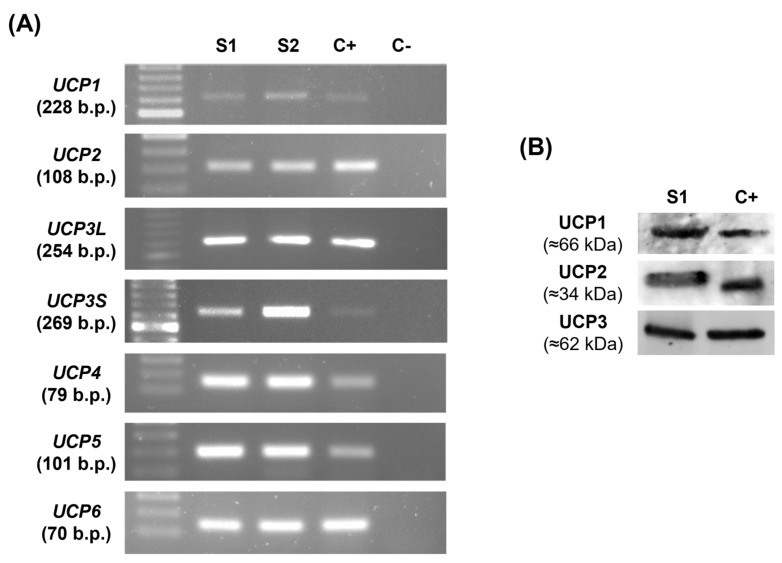
Identification of mitochondrial uncoupling proteins (UCPs) homologs (UCP1-6) mRNA and protein (UCP1-3) expression in human spermatozoa. (**A**) Representative RT-PCR of *UCP1*, *UCP2*, *UCP3L*, *UCP3S*, *UCP4*, *UCP5*, and *UCP6* mRNA expression in human spermatozoa. Samples from two different individuals (S1 and S2) were used and cDNA from human peripheral blood leukocytes (C+) was used as a positive control. (**B**) Representative blots of UCP1, UCP2, and UCP3 protein expression in human spermatozoa (S1). Mouse kidney was used as a positive control (C+).

Few studies focused on studying UCPs expression and function in human spermatozoa. In this study, we screened for mRNA expression of known UCPs homologs (*UCP1-6*) by human spermatozoa. Human peripheral blood leukocytes (PBL) were used as a positive control. *UCP1*, *UCP2*, *UCP3S* (UCP3 short isoform), *UCP3L* (UCP3 long isoform), *UCP4*, *UCP5*, and *UCP6* mRNA were identified in human spermatozoa (Figure 2A). Protein expression of UCP1, UCP2, and UCP3 in human spermatozoa was also confirmed by Western Blot (Figure 2B). Protein expression of UCP4, UCP5, and UCP6 was not tested as there were no validated antibodies commercially available. Our results show a blot band representative of UCP2 at the predicted molecular weight (≈34 kDa). However, UCP1 and UCP3 blot bands presented a higher molecular weight (≈66 kDa and ≈62 kDa, respectively), which could be suggestive of dimerization, a process that has been previously reported [26].

### 3.2. UCP1, UCP2, and UCP3 Are Mainly Located at the Equatorial and Acrosome Region of the Head of Human Spermatozoa

**Figure 3:**
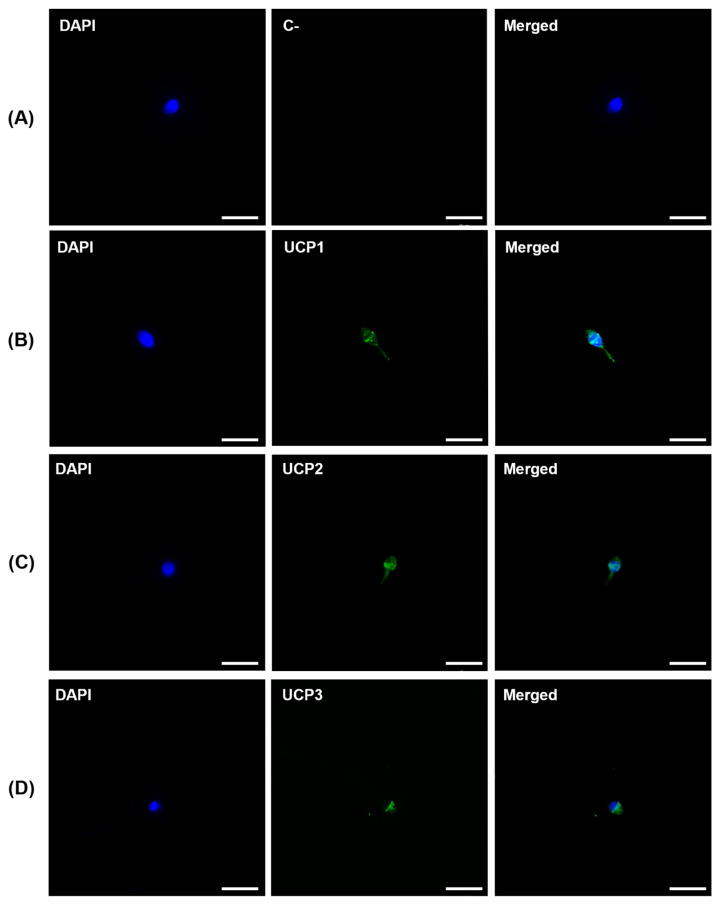
Immunofluorescence staining for UCP1, UCP2, and UCP3 protein expression in human spermatozoa. (**A**) Negative control. Immune labeling of UCP1 (**B**), UCP2 (**C**), and UCP3 (**D**) was observed in human spermatozoa. Scale bar—10 µm.

UCP1, UCP2, and UCP3 location in human spermatozoa were evaluated by immunofluorescence staining (Figure 3). Notably, all UCPs homologs studied were found to be expressed outside the midpiece, where the mitochondria are mainly present. UCP1 (Figure 3B) and UCP2 (Figure 3C) were found to be expressed at the head, with strong staining in the equatorial region and lighter staining in the acrosome region and midpiece. UCP3 (Figure 3D) was only found to be expressed at the head, specifically at the equatorial and acrosome region. No fluorescent staining was observed in the negative control (Figure 3A).

### 3.3. UCPs Inhibition Induces Motility Loss without Compromising Viability of Human Spermatozoa

**Figure 4:**
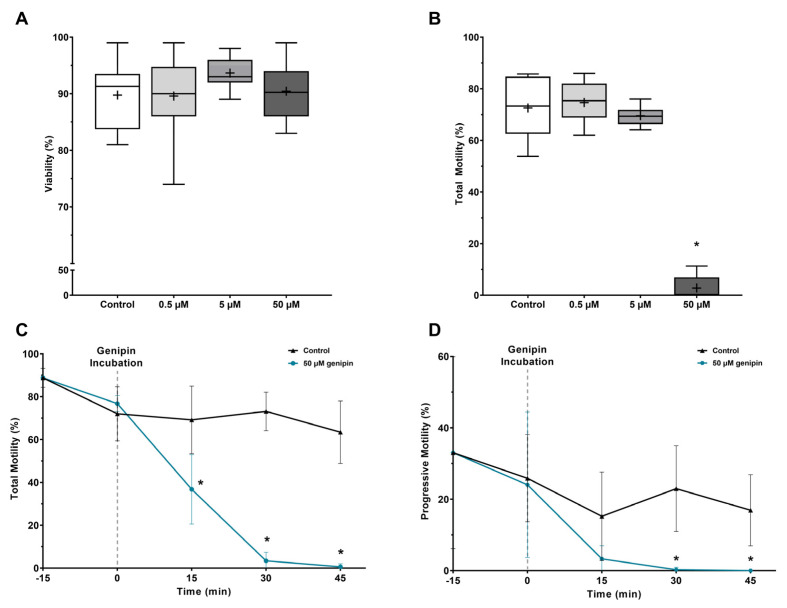
Evaluation of human spermatozoa viability and motility after UCPs inhibition by increasing concentrations of genipin (0.5, 5, and 50 µM) for 3 h. (**A**) Human spermatozoa viability was determined by eosin–nigrosin staining (n = 10). (**B**) Effect of UCPs inhibition on total motility of human spermatozoa (n = 10). (**C**) Evaluation of the time of action of 50 µM genipin on total and (**D**) progressive motility (n = 6). Results are expressed as Tukey’s boxplot (median, 25th to 75th percentiles ± 1.5 IQR) (**A**,**B**) or mean ± SD (**C**,**D**). +−Represents group average. Significantly different results are expressed as: *−Relative to the control group.

To study the role of UCPs in human spermatozoa physiology we used an UCPs inhibitor, genipin. The percentage of viable spermatozoa following UCPs inhibition by genipin (0, 0.5, 5, and 50 µM) for 3 h was analyzed through eosin–nigrosin staining (Figure 4A). No differences were observed between all experimental groups (90 ± 7%, 94 ± 3%, and 90 ± 5% after treatment with 0.5, 5, or 50 µM genipin, respectively) when compared to the control group (90 ± 6%).

Interestingly, we observed that UCPs inhibition greatly affected spermatozoa motility (Figure 4B). UCPs inhibition with the highest genipin concentration (50 µM) greatly decreased spermatozoa total motility (3 ± 5%) as compared to the control group (73 ± 11%, *p* < 0.001). A decrease in total motility was not observed in the remaining experimental groups (75 ± 8% and 70 ± 4% after treatment with 0.5 and 5 µM genipin, respectively) as compared to the control group.

We further evaluated the time of action of UCPs inhibition by 50 µM genipin in spermatozoa total and progressive motility. For that purpose, spermatozoa were incubated in modified BWW media for 15 min and then genipin was added. Total (Figure 4C) and progressive motility (Figure 4D) were assessed every 15 min for a total of 45 min. A decrease in total motility was observed as early as after 15 min of incubation (37 ± 16%) when compared to the control group (69 ± 16%, *p* = 0.013), whereas a tendency for a decrease of progressive motility (3 ± 4%) was also observed within the same time interval when compared to the control group (15 ± 12%, *p* = 0.07). A significant motility loss conferred by UCPs inhibition was achieved at 30 min (3 ± 4% and 0 ± 1% for total and progressive motility, respectively) whereas spermatozoa from the control group remained motile (73 ± 9%, *p* < 0.001; 23 ± 12%, *p* = 0.006 for total and progressive motility, respectively). Virtually full total (1 ± 1%) and full progressive motility loss (0 ± 0%) was observed at 45 min. Spermatozoa from the control group sustained its motility (63 ± 15%, *p* < 0.001; 17 ± 10%, *p* = 0.009 for total and progressive motility, respectively).

### 3.4. Human Spermatozoa Motility Loss Due to UCPs Inhibition Is Not Recovered by Incubation with Albumin or Theophylline 

**Figure 5:**
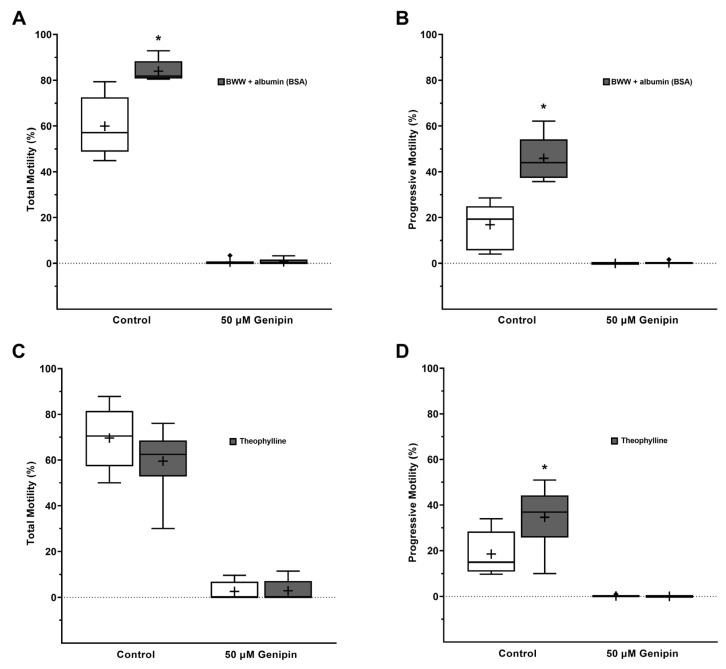
Evaluation of motility loss reversibility conferred by UCPs inhibition with 50 µM genipin. (**A**) Total and (**B**) progressive motility of genipin-treated spermatozoa upon wash and 45 min incubation in modified BWW media supplemented with albumin (n = 6). (**C**) Total and (**D**) progressive motility analysis of genipin-treated spermatozoa upon 20 min incubation with theophylline (SpermMobil) (n = 6). Results are expressed as Tukey’s boxplot (median, 25th to 75th percentiles ± 1.5 IQR). +−Represents group average. Significantly different results are expressed as: *−Relative to genipin-treated group.

After observing that UCPs inhibition led to complete motility loss, we aimed to study whether this condition was reversible. For that purpose, we washed and incubated genipin-treated spermatozoa in modified BWW media supplemented with albumin. Albumin is reported to form complexes with genipin, thus decreasing its physiological effects [22]. After 45 min of incubation, total (Figure 5A) and progressive motility (Figure 5B) were analyzed. An increase in total (84 ± 5%) and progressive motility (46 ± 10%) was observed in the control group as compared to pre-washing and albumin incubation parameters (60 ± 13%, *p* = 0.030; 17 ± 10%, *p* = 0.001 for total and progressive motility, respectively). Nevertheless, no improvement in total (1 ± 1%) or progressive motility (0 ± 1%) was observed as compared to pre-washing and albumin incubation parameters (1 ± 1%; 0 ± 0% for total and progressive motility, respectively).

To further evaluate the reversibility of genipin effects, theophylline (SpermMobil), a cAMP activator, was also used. After 20 min incubation, total (Figure 5C) and progressive motility (Figure 5D) were analyzed. Although no changes in total motility (60 ± 16%) were observed, progressive motility (35 ± 14%) increased in the control group, as compared to pre-treatment parameters (70 ± 15%; 19 ± 10%, *p* = 0.036 for total and progressive motility, respectively). Supporting the previous results, no improvement in total (3 ± 5%) or progressive motility (0 ± 0%) was observed when compared to the pre-treatment parameters (3 ± 4%; 0 ± 0% for total and progressive motility, respectively). Taken together, these results suggest that UCPs inhibition induced by genipin leads to irreversible motility loss.

### 3.5. UCP3 Relative Expression in Spermatozoa Does Not Differ between Normozoospermic and Asthenozoospermic Men

**Figure 6:**
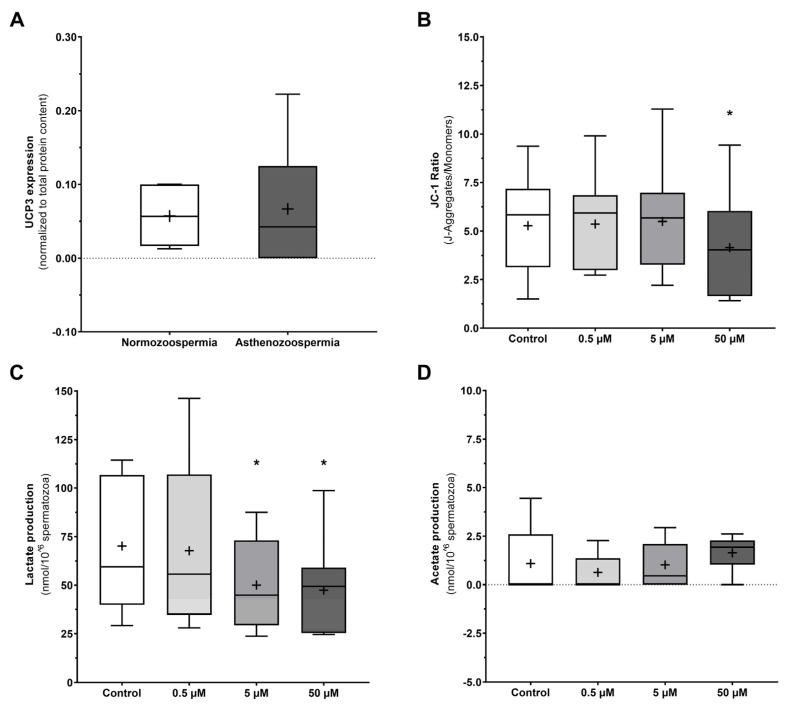
UCP3 expression, mitochondrial membrane polarization, and metabolic profile of human spermatozoa. (**A**) UCP3 relative expression in spermatozoa from normozoospermic versus asthenozoospermic men. (**B**) Evaluation of mitochondrial membrane potential using the JC-1 dye after inhibition of UCPs by increasing concentrations of genipin (0.5, 5, and 50 µM) for 3 h. (**C**) Lactate and (**D**) acetate production by human spermatozoa after UCPs inhibition by increasing concentrations of genipin (0.5, 5, and 50 µM) for 3 h. Results are expressed as Tukey’s boxplot (median, 25th to 75th percentiles ± 1.5 IQR). +−Represents group average. Significantly different results are expressed as: *−Relative to the control group.

Taking into consideration that 50 µM genipin induced near total motility loss, we hypothesize that these effects could be mainly mediated by UCP3 inhibition (IC_50_ value previously reported as 42 ± 12 µM [18]). To disclosure whether spermatozoa with decreased motility had altered UCP3 expression or dysfunction, we compared UCP3 expression in spermatozoa of asthenozoospermic (low motility, <40%) and normozoospermic (normal sperm parameters) men by Western Blot (Figure 6A). Although we were not able to detect UCP3 in some samples from asthenozoospermic men, no differences concerning mean UCP3 expression were observed when spermatozoa of asthenozoospermic (0.076 ± 0.084 arbitrary units) were compared to those of normozoospermic (0.057 ± 0.038 arbitrary units) men, suggesting that a potential dysfunction leading to motility loss is unlikely to be mediated by altered UCP3 expression.

### 3.6. UCPs Inhibition Decreases Mitochondrial Membrane Potential and Lactate Production in Human Spermatozoa

To further disclose the molecular mechanisms that lead to motility loss conferred by UCPs inhibition, we sought to evaluate the mitochondrial membrane potential using the fluorogenic dye JC-1 (Figure 6B). At the highest concentration of genipin (50 µM) there was a decrease in the JC-1 ratio (4.147 ± 2.585 J-aggregates/monomers) when compared to the control group (5.282 ± 2.509 J-aggregates/monomers, *p* = 0.020), indicating mitochondrial depolarization and decreased mitochondrial activity. No differences were observed when the groups incubated with lower genipin concentrations (5.366 ± 2.394 and 5.497 ± 2.699 J-aggregates/monomers for 0.5 and 5 µM, respectively) were compared to the control group.

We also analyzed the culture media by ^1^H-NMR spectra to assess if UCPs inhibition leading motility loss had an impact on the human spermatozoa metabolic signature. Lactate (Figure 6C) and acetate (Figure 6D) were the main metabolites identified and quantified. We observed that both the 5 and 50 µM of genipin led to lower lactate production (47.43 ± 23.33 and 50.07 ± 23.61 nmol/10^6^ spermatozoa for 5 and 50 µM, respectively) as compared to the control group (70.14 ± 34.62 nmol/10^6^ spermatozoa, *p* = 0.025 and *p* = 0.010 for 5 and 50 µM, respectively). No differences were observed when the group treated with the lower genipin concentration (67.73 ± 41.73 nmol/10^6^ spermatozoa) was compared to the control group. No differences were observed concerning acetate production (0.63 ± 0.89; 1.03 ± 1.14; 1.64 ± 0.93 nmol/10^6^ spermatozoa for 0.5, 5, and 50 µM, respectively) as compared to the control group (1.10 ± 1.63 nmol/10^6^ spermatozoa).

### 3.7. UCPs Inhibition Does Not Increase Total ROS Production by Human Spermatozoa

**Figure 7:**
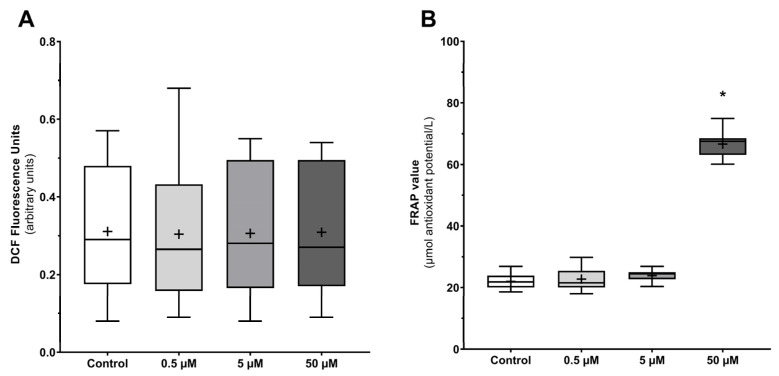
Culture media total antioxidant capacity and endogenous ROS production by human spermatozoa after UCPs inhibition by increasing concentrations of genipin (0.5, 5, and 50 µM) for 3 h. (**A**) Evaluation of endogenous ROS production using the CM-H_2_DCFDA dye. (**B**) Analysis of the culture media antioxidant potential through ferric antioxidant power (FRAP) assay. Results are expressed as Tukey’s boxplot (median, 25th to 75th percentiles ± 1.5 IQR). +−Represents group average. Significantly different results are expressed as: *−Relative to the control group.

Since UCPs dysfunction is closely linked to redox unbalance, we aimed to evaluate total intracellular ROS production using the fluorogenic dye CM-H_2_DCFDA (Figure 7A). Interestingly, there were no significant differences concerning ROS production by spermatozoa after genipin exposure at all studied concentrations (0.304 ± 0.189; 0.306 ± 0.172; 0.309 ± 0.171 DCF fluorescence units after treatment with 0.5, 5, and 50 µM, respectively) as compared to the control group (0.311 ± 0.162 DCF fluorescence units). In addition, culture media antioxidant potential was tested using the FRAP assay (Figure 7B). We observed that the culture media supplemented with genipin at the highest concentration (50 µM) had an increased antioxidant capacity (66.63 ± 4.11 µmol antioxidant potential/L) as compared to the control group (22.00 ± 2.52 µmol antioxidant potential/L, *p* < 0.001), denoting the genipin antioxidant properties. No differences were observed when the groups treated with lower genipin concentrations (22.77 ± 3.63 and 23.90 ± 2.06 µmol antioxidant potential/L for 0.5 and 5 µM, respectively) were compared to the control group.

## 4. Discussion

UCPs are mitochondrial proteins known for their activity as regulators of ROS production and oxidative stress. Although initially identified as adipose tissue-specific proteins, it is currently known that UCPs are present almost throughout the human body. However, limited data are available concerning its expression and function in the human male reproductive system, including spermatozoa. For instance, only UCP2 has been reported to be present in human spermatozoa [27]. Although studies with mRNA microarray screening also predicted the expression of the newly identified UCP5 and UCP6 in the testis, no further studies were conducted since [28]. In this work, we aimed to identify the expression and characterize the function of UCPs in human spermatozoa. For this purpose, we screened mRNA expression of UCP1-6 homologs, and, to the best of our knowledge, we were able to identify for the first time the transcripts of all six UCP homologs—*UCP1*, *UCP2*, *UCP3* (both short and long isoform, *UCP3S* and *UCP3L*)*, UCP4*, *UCP5*, and *UCP6.* Due to its function, the expression of all UCP homologs in human spermatozoa suggests its possible involvement in the complex and finely regulated role of ROS production and ROS-dependent signalling pathways, essential for several sperm functions, including capacitation and also acrosome reaction [29]. We also confirmed the presence of UCP1, UCP2, and UCP3 proteins. Interestingly, the cellular location of these UCPs provided surprising results. We observed that UCP1, UCP2, and UCP3 are also expressed outside its classic location within the mitochondrial inner membrane, found at the midpiece of spermatozoa. While UCP1 and UCP2 were also found in the midpiece, UCP1, UCP2, and UCP3 were found to be mainly expressed in the equatorial region of the head of human spermatozoa. Interestingly, we found that UCP3 was specifically expressed in the head. The expression of UCP2 in the midpiece of human spermatozoa was previously reported by Wang et al. [27], but the expression of UCPs outside the mitochondria is herein described for the first time. The equatorial segment is a unique region found at the head of mammalian spermatozoa. It differs from the plasma membrane that covers the anterior and post-acrosomal domains in terms of both structure and function [30], as it is reported to express several specific proteins and ionic channels [31,32]. Since it was shown to be the region of fusion with the oocyte membrane in mammalian species, the equatorial segment function has attracted particular research interest. Spermatozoa only acquire fusion competence after acrosome reaction, the last maturational stage to acquire fertilizing ability, in which the acrosomal proteases are released to activate fusion proteins [33]. In recent work, Ren et al. showed that the acrosomal matrix contains condensed mitochondria, which arise from progressive matrix contraction of orthodox mitochondria during germ cells meiosis [34]. These condensed mitochondria were reported to exhibit increased respiration and higher levels of respiratory enzymes and supercomplexes. Therefore, we hypothesize that UCPs expression at the head of human spermatozoa could potentially pinpoint the presence of those mitochondria. In addition, mitochondria are the main source of ROS and acrosome reaction is regulated by ROS and ROS-dependent signalling pathways [29]. Ichikawa et al. observed a strong negative correlation between ROS concentration and acrosome reaction [35]. In another study, Aitken et al. observed that hydrogen peroxide promoted sperm penetration of zona-free hamster eggs, which would not occur in the presence of catalase [36]. Griveau et al. also observed that increased levels of O_2_^-^ radicals induced acrosome reaction [37]. Thus, ROS regulation and signalling seem to be essential for acrosome reaction, and due to its location at the acrosomal region, UCPs seem to be potential mediators in the regulation of this mechanism. Nevertheless, future studies are needed to disclose the role of UCPs in the acrosome reaction.

The role and function of UCPs are shrouded in mystery as conflicting results are found in the literature. To further characterize UCPs function in human spermatozoa, we used the UCPs inhibitor genipin. This molecule was firstly described as a selective UCP2 inhibitor but was lately demonstrated to be also able to inhibit UCP1 and UCP3 dose-dependently, without reducing protein expression [18]. The chosen doses of genipin were based on studies previously reported in the literature. The lower doses of genipin (0.5 and 5 µM) were chosen according to the mitochondrial and metabolic effects observed in pancreatic β-cells by Zhang et al. [38]. In this study, these authors observed that small doses of genipin (0.5 and 5 µM, which are, respectively, approximately 200 and 20 times lower than the reported IC_50_ for UCP2 inhibition—94 ± 24 µM [18]) increased mitochondrial membrane potential and ATP levels in pancreatic β-cells, thus stimulating insulin secretion. The highest concentration used in our study (50 µM) was chosen based on the IC_50_ value reported for UCP3 inhibition (42 ± 12 µM) [18]. We performed a pilot study (data not shown) using an even higher concentration (100 µM, based on the IC_50_ value reported for UCP2 inhibition) but since total loss of motility was observed with 50 µM of genipin, this concentration was chosen as the highest concentration. Surprisingly, genipin at 50 µM induced a motility loss without compromising spermatozoa viability. These effects on spermatozoa motility were shown to be relatively fast and enduring, as motility loss was achieved in 30 min and persisted for over 3 h. As motility loss was achieved at 50 µM, we hypothesize that these effects potentially occurred mainly due to UCP3 inhibition, although inhibition of the remaining UCPs could contribute to a smaller extent. Interestingly, UCPs inhibition conferred by genipin seems to be irreversible. At first, we washed and incubated spermatozoa in modified BWW media supplemented with albumin. Albumin is required for spermatozoa capacitation and increased motility [23], but it was excluded from our experiments since it triggers the formation of complexes with genipin, thus decreasing its effects [22]. Although total and progressive motility increased when spermatozoa were washed and incubated with albumin, the motility loss conferred by genipin was irreversible. Similar results were observed when spermatozoa were washed and incubated with theophylline (SpermMobil). Theophylline is a methylxanthine, a class of compounds that act as phosphodiesterases (PDEs) inhibitors [39]. Cyclic nucleotide PDEs are responsible for the degradation of cAMP, a molecule that is central in the process of capacitation, and therefore, its inhibition increases cAMP levels which promotes progressive motility [40]. Although we observed increased progressive motility upon exposure to theophylline in the control group, genipin-incubated spermatozoa did not recover from the loss of motility. Taken together, these results suggest that the motility loss induced by the inhibition of UCPs by genipin is potentially permanent.

Since the loss of motility induced by genipin is potentially mediated by UCP3 inhibition, we sought to evaluate if UCP3 expression was altered in the spermatozoa of men with asthenozoospermia. Interestingly, no differences in UCP3 expression were observed when spermatozoa from asthenozoospermic and normozoospermic men were compared, which suggests that an altered function rather than altered expression could be associated with decreased motility. The motility of spermatozoa is supported by both glycolysis and mitochondrial function [41]; thus, we hypothesized that mitochondrial function could be compromised or at least altered. Indeed, decreased mitochondrial membrane potential was observed at the highest concentration of genipin (50 µM) which led to complete sperm motility loss. Since UCP3 was only found to be expressed at the head, it is unlikely that the observed decrease in mitochondrial membrane potential is due to its inhibition. However, UCP1 and UCP2 were found to be expressed in the midpiece, where mitochondria are located, thus the decreased mitochondrial membrane may potentially occur due to its inhibition. Both UCP1 and UCP2 are well-known modulators of the mitochondrial membrane potential in addition to cellular metabolism [42,43]. Thus, we also studied the metabolic profile of human spermatozoa upon UCPs inhibition and observed decreased lactate production. Lactate is the main metabolite released by human spermatozoa, as a product of glycolysis [44]. Notably, the inhibition of UCPs decreased lactate production at both 5 and 50 µM, even though no motility alterations were observed with the lower concentration. Acetate production, on the other hand, was unaltered under all studied conditions. A decrease in lactate production due to UCPs inhibition has been reported in other cell types. Vozza et al. observed that the silencing of *UCP2* resulted in decreased lactate production by HepG2 cells, leading to the suggestion that UCPs favour glycolytic pathways through the modulation of Krebs cycle intermediates levels [45]. Similar results were observed in rat cardiomyocytes, in which *Ucp2* silencing decreased intracellular lactate levels [46]. We hypothesize that the decreased lactate production is linked to decreased ATP production through glycolysis, which in turn would reduce spermatozoa motility. Taken together, our data suggest that UCPs are metabolic modulators of human spermatozoa, although the UCP-mediated molecular mechanisms remain to be elucidated.

Dysfunction of UCPs is positively related to oxidative stress, which is a known major cause of male infertility. While UCP1 is usually related to thermogenesis, UCP2 and UCP3 had been mainly studied as regulators of ROS production. Interestingly, UCP2 and UCP3 do not display a clear uncoupling activity, being activated due to ROS overproduction through a negative feedback mechanism [47]. It is reported that UCP2 and UCP3 overexpression reduces ROS production [48,49,50], whereas its knockout increases oxidative stress [51,52]. Thus, UCPs are hypothesized to take part in the complex and finely regulated network that maintains cellular ROS balance. The mechanisms, however, are still fairly unknown. In this work, no differences concerning intracellular ROS production upon UCPs inhibition were observed. This finding could be explained by decreased mitochondrial activity. Mitochondria are the main source of ROS in human spermatozoa [53]. In our previous study, we demonstrated that increased ROS production occurs in spermatozoa submitted to *in vitro* capacitation conditions after mitochondrial activation [23]. As genipin decreases mitochondrial activity, an increased ROS production will not occur, suggesting that UCPs inhibition could, potentially, inhibit capacitation. This hypothesis, however, needs confirmation in future studies. Simultaneously, through the FRAP assay, we observed that genipin has antioxidant properties. The antioxidant properties of genipin have been reported before in the literature [54,55]. Interestingly, both the antioxidant properties and decreased mitochondrial activity were observed at 50 µM, but ROS levels remained unaltered whereas a decrease could be expected. These results suggest that basal ROS levels are maintained, further suggesting a finely regulated and complex network of ROS and ROS-dependent signaling pathways that regulates spermatozoa function.

Some limitations of this study should be considered. One of the limitations of this study is the small sample size and thus, studies with larger sample sizes should be conducted to confirm our hypotheses. In addition, the use of genipin as an UCPs inhibitor has its own limitations. Although initially reported as a selective UCPs inhibitor, it is currently known that genipin, at 50 µM, is also able to inhibit the mitochondrial complex III (CIII) by 30% [18]. The inhibition of CIII can be reflected in the decreased mitochondrial membrane potential and can also be related to the decreased motility due to inhibition of oxidative phosphorylation and decreased ATP production. Overall, we hypothesize that it is not the main contributor to the observed motility loss. Spermatozoa are known to have a higher glycolytic activity as compared to the Krebs cycle and oxidative phosphorylation [41]. In human spermatozoa, glycolysis is hypothesized to be the main source of ATP, which occurs mainly in the tail to sustain motility [56]. Additionally, the well-known CIII inhibitor antimycin A was described as being more potent than genipin [18], and significantly decreased but not conferred loss of motility in spermatozoa [57]. Thus, we conclude that UCPs inhibition promoted by genipin is the main cause for the observed motility loss.

## 5. Conclusions

In conclusion, our results demonstrate for the first time, to the best of our knowledge, the expression of UCP1-6 homologs in human spermatozoa. Interestingly, UCP1, UCP2, and UCP3 were found to be expressed at the equatorial segment of the head, besides the expression of UCP1 and UCP2 at the midpiece, where the mitochondria are located. UCPs are major regulators of spermatozoa motility, as its inhibition by genipin leads to a relatively rapid and irreversible motility loss. Interestingly, UCPs inhibition in human spermatozoa promotes motility loss without compromising its viability. Taking into consideration the concentration of genipin used, we propose that the motility loss occurs due to UCP3 inhibition, although the inhibition of other homologs could also contribute to a smaller extent. Interestingly, no altered UCP3 expression was found in spermatozoa from asthenozoospermic men, suggesting there are other causes for the dysfunction. UCPs are also modulators of human spermatozoa metabolism, and potentially of capacitation and acrosome reaction, although future studies are needed to address these hypotheses. Since silencing in human spermatozoa cannot be performed, the discovery and characterization of selective UCP inhibitors are required. Taken together, the discovery and characterization of UCPs’ role in human spermatozoa physiology can shed some light on spermatozoa bioenergetics and the role of ROS and ROS-related pathways for capacitation and acrosome reaction. The potential role of UCPs (dys)function in male oxidative stress-related infertility and asthenozoospermia should also be highlighted, opening the path for future lines of investigation.

## Figures and Tables

**Table 1:** Oligonucleotides and cycling conditions for RT-PCR amplification of mitochondrial uncoupling proteins (UCPs) homologs.

Gene	Sequence 5′-3′	Annealing Temperature	Number of Cycles
*UCP1* (NM_021833.5)	FWD: GCTCCAGGTCCAAGGTGAAT RVS: TTGCTTCCTAAACTAGGTGCTG	59 °C	40
*UCP2* (NM_001381943.1)	FWD: GAAGCCTCTACAATGGGCTGG RVS: CAGAGCCCTTGGTGTAGAACTG	63 °C	30
*UCP3S* (NM_022803.3)	FWD: CAACCTGGGATGTAGCGGTG RVS: GAGCGTGTGGAGACAGTGAG	60 °C	25
*UCP3L* (NM_003356.4)	FWD: TGGAACGTGGTGATGTTCGTA RVS: AGACCAGAATCCCTCCTCCT	60 °C	33
*UCP4* (NM_004277.5)	FWD: CGCACAGCTCTAGGGATCAT RVS: CGTGTCTGTAAATGGCGGGT	60 °C	33
*UCP5* (NM_001282195.2)	FWD: GACTTTCCCTGTGGACCTTACC RVS: GCGCATGGAACATCCCTCTAT	60 °C	33
*UCP6* (NM_001010875.4)	FWD: ACCCTGTTGATGTTGTGAGG RVS: TAGCCAGAACATCTGCCATCTC	63 °C	33

## Data Availability

The data presented in this study are available on request from the corresponding author. The data are not publicly available due to privacy and ethical restrictions.
